# Inhibin secretion in women with the polycystic ovary syndrome before and after treatment with progesterone

**DOI:** 10.1186/1477-7827-9-59

**Published:** 2011-04-29

**Authors:** Konstantinos Dafopoulos, Christos Venetis, Christina I Messini, Spyros Pournaras, George Anifandis, Antonios Garas, Ioannis E Messinis

**Affiliations:** 1 Department of Obstetrics and Gynaecology, Medical School, University of Thessalia, Larissa, Greece; 2 Department of Microbiology, Medical School, University of Thessalia, Larissa, Greece

## Abstract

**Objectives:**

It has been suggested that inhibin secretion is altered in women with the polycystic ovary syndrome (PCOS). However, the contribution of a preceding luteal phase has not been taken into account. The aim of the present study was to investigate whether progesterone in the context of a simulated luteal phase affects basal and FSH-induced inhibin secretion in women with PCOS and elevated LH.

**Methods:**

Ten women with PCOS and 8 normally cycling women participated in an experimental procedure (Exp) involving the administration of a single injection of recombinant FSH (450 IU sc). In the women with PCOS, the procedure was performed before (Exp 1) and after a 20-day treatment with progesterone (Exp 2), while in the normal women on day 2 of the cycle (Exp 3). Inhibin A and B levels were measured in blood samples taken before and 24 hours after the FSH injection.

**Results:**

Basal LH levels were significantly higher and inhibin A levels were significantly lower in the PCOS group compared to the control group, while inhibin B levels were comparable in the two groups. In the PCOS group, after treatment with progesterone inhibin A and LH but not inhibin B levels decreased significantly (p < 0.05). After the FSH injection, inhibin A and B levels increased significantly in the women with PCOS (Exp 1 and Exp 2) but not in the control women (Exp 3).

**Conclusions:**

In women with PCOS, as compared to control women, the dissimilar pattern of inhibin A and inhibin B secretion in response to FSH appears to be independent of a preceding simulated luteal phase. It is possible that compared to normal ovaries, the PCOS ovaries are less sensitive to endogenous LH regarding inhibin A secretion and more sensitive to exogenous FSH stimulation in terms of inhibin A and inhibin B secretion.

## Background

Data regarding inhibin levels in the circulation of women with polycystic ovary syndrome (PCOS) are conflicting. In particular, when compared to controls inhibin B concentrations have been found to be either increased [1,2] or similar [3-9]. On the other hand, most studies [3,7,10], but not all [2] have suggested that inhibin A levels are lower in women with PCOS than in control women. It has been suggested that in normal women, the intercycle rise of FSH is responsible for the increased secretion of inhibin B coming from the small antral follicles in the early follicular phase, while the midcycle LH increase stimulates inhibin A secretion coming from the pre-ovulatory follicle [11]. However, in women with PCOS, the role of gonadotrophins in the regulation of inhibin secretion is less clear and is based on data mainly derived from experiments involving the exogenous administration of FSH [2,9,12]. Even so, the data are scanty showing differences between PCOS and controls regarding the pattern of inhibin B secretion in response to a single or multiple doses of FSH [2,9,12]. On the other hand, inhibin A levels showed a similar pattern of increase in PCOS and in control women [2,12].

The reason for the differences between PCOS and normally cycling women regarding the inhibin response to FSH has not yet been defined. Previous experiments investigating the dynamic response of inhibin to a single dose of FSH were performed in PCOS women at a random day or after exclusion of recent ovulation, i.e. at a time far beyond the luteal phase, while in normally cycling women in the early to mid follicular phase [9,12]. Therefore, it is not known if the above differences in inhibin secretion are related to the lack of progesterone in PCOS due to chronic anovulation. It has been shown that exogenous progesterone reduces elevated circulating LH levels in women with PCOS [13], while LH may affect inhibin A secretion in normally cycling women [11].

The present study was undertaken to test the hypothesis if the distinct pattern of inhibin secretion under the stimulation by FSH in women with PCOS results from the lack of the luteal phase. The present experiments were performed in women with PCOS and elevated LH after the exogenous administration of progesterone, as a surrogate of previous ovulation and luteal phase function.

## Methods

### Subjects

Ten women with PCOS (aged 24.5 ± 2.1 years) and 8 women with normal menstrual cycles (aged 31.5 ± 1.0 years), which served as controls, volunteered for the study and gave written informed consent. The body mass index (BMI) of women with PCOS was 26.0 ± 2.5 kg/m^2 ^and that of the normal women 21.0 ± 1.1 kg/m^2^. The difference was not significant. Institutional Review Board approval of the study was obtained. Polycystic ovary syndrome was diagnosed according to the revised 2003 criteria [14]. In particular, all women had polycystic ovaries by ultrasound and amenorrhoea or oligomenorrhoea. In addition to that, all women with PCOS had elevated LH with LH to FSH ratio greater than 2. Exclusion criteria were normal LH, normal cycles, obesity (BMI > 30 kg/m^2^) and any medical or hormonal treatment during the last six months before the women entered the study. The women with PCOS were recruited at an outpatient clinic of gynaecological endocrinology. A total of 35 women with PCOS were screened and 10 were finally included fulfilling the above criteria. Sample size calculation was based on the data of a previous study [12].

All women participated in the same experimental procedure (Exp) involving the sc injection of a single dose 450 IU recombinant FSH (Puregon 150 IU; Organon Hellas, Athens, Greece) (0900 h). The dose of exogenous FSH was based on our previous studies [15-17]. The women with PCOS were investigated in two experimental procedures, i.e. before (Exp 1) and after a 20-day treatment with progesterone (Exp 2). The Exp 1 was performed on a random day at least 15-20 days after a spontaneous period and after ovarian quiescence was confirmed by ultrasonography and serum estradiol and progesterone measurement. The administration of progesterone started the day after the end of the Exp 1. The Exp 2 was performed on the day following the end of the 20-day treatment with progesterone (Utrogestan capsules, 100 mg; Faran, Athens, Greece) at the oral dose of 300 mg/day. Although the length of the normal luteal phase is no more than 16 days [18], in the present study progesterone was given to the PCOS women for 20 days in order to enhance the progestational effect in these patients who only occasionally are exposed to this steroid due to chronic anovulation. In the normally cycling women (Exp 3), the dose of 450 IU FSH was given sc on day 2 of the cycle.

In each experiment, two blood samples were taken, one before and a second sample 24 hours after the FSH injection. All blood samples were centrifuged at 1000 g for 15 min and serum was stored at -20°C until assayed. Serum FSH, LH, inhibin A and inhibin B were measured in all blood samples.

### Assays

Measurement of FSH and LH in serum was performed using a Chemiluminescent Microparticle Immunoassay (Architect FSH and Architect LH respectively; Abbott Laboratories, Illinois, USA). The results are expressed as IU/l for FSH and LH. Inhibin A and inhibin B were measured using an enzyme-linked immunosorbent assay (Inhibin A, Active^® ^ELISA and Inhibin B, Active^® ^ELISA, Diagnostic Systems Laboratories, Texas, USA). The results are expressed as pg/ml. The lower limits of detection for FSH, LH, inhibin A and inhibin B were 0.05 IU/l, 0.07 IU/l, 1 pg/ml and 7 pg/ml respectively. Inter- and intra-assay coefficients of variation were 3.1 and 3.4%, 2.0 and 3.4%, 7.8 and 3.6% and 7.2 and 5.6% respectively.

### Statistical analysis

Hormone values were normally distributed (one sample Kolmogorov-Smirnov test). Statistical analysis was performed by paired t-test, one-way analysis of variance (ANOVA) followed by Bonferroni post hoc testing. All values are expressed as means ± SEM. An α-level of 0.05 was used to determine statistical significance. The statistical software package used was SPSS 15.0.

## Results

**Figure 1:**
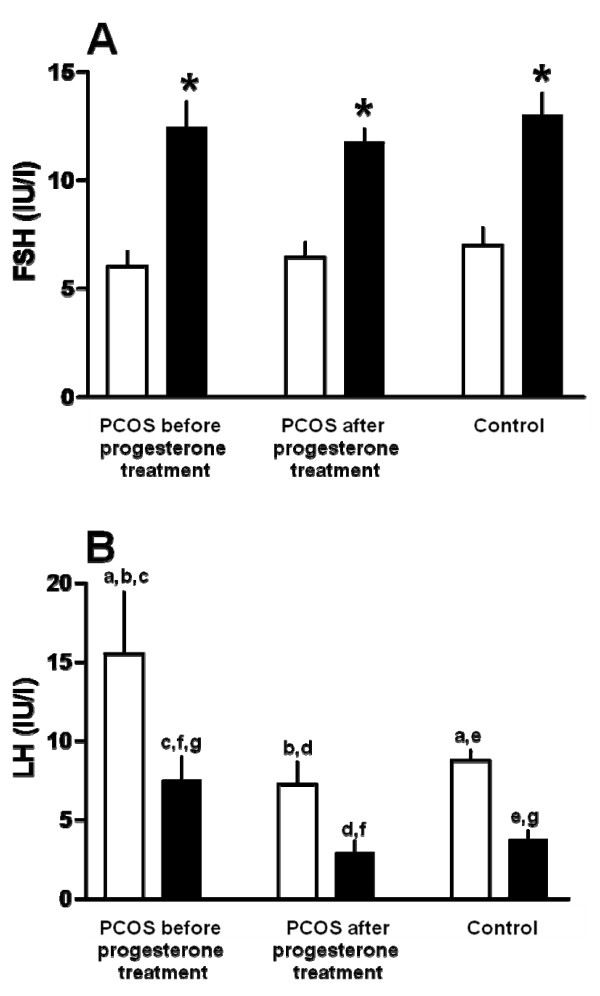
**Serum FSH and LH levels**. (A) FSH and (B) LH levels before (white bars) and 24 hours after a single injection of recombinant FSH (450 IU sc) (black bars) in 10 women with PCOS and 8 normally cycling women (control). The FSH injection was given to the women with PCOS twice, i.e. first 15-20 days after a spontaneous period (before progesterone treatment) and second after a 20-days treatment with progesterone per os (after progesterone treatment). The FSH injection was given to the normally cycling women on cycle day 3. After the FSH injection, serum FSH levels increased and serum LH levels decreased significantly. A. *p < 0.05, comparison with corresponding values before the FSH injection. B. a,b,c,d,e,f,g p < 0.05.

Except inhibin values, hormone values following the FSH injection have been reported in more details elsewhere [19]. Serum estradiol and progesterone concentrations did not differ significantly between PCOS and controls [19]. Before the FSH injection, serum FSH levels did not differ significantly between the three experiments (Figure 1A). Following the FSH injection, serum FSH concentrations increased significantly in all three experiments (p < 0.05), with no significant difference between them (Figure 1A). Serum LH levels were before the FSH injection significantly higher in the women with PCOS (Exp 1) as compared to the control women (Exp 3) (p < 0.05) but treatment with progesterone reduced significantly the LH values to the normal range (Exp 2, Figure 1B). After the FSH injection, LH values decreased in all experiments (p < 0.05), that in the PCOS women were still significantly higher than in the controls (p < 0.05) (Figure 1B). At the same time, LH values were significantly higher in the PCOS women before as compared to after progesterone administration.

**Figure 2:**
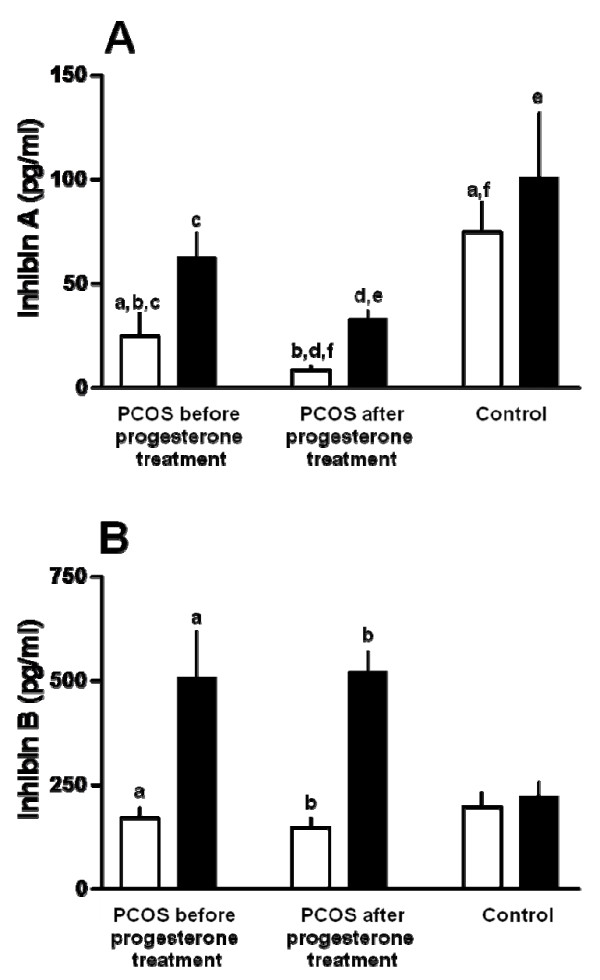
****Serum inhibin A and B levels**. **(A) inhibin A and (B) inhibin B levels before (white bars) and 24 hours after a single injection of recombinant FSH (450 IU sc) (black bars) in 10 women with PCOS and 8 normally cycling women (control). The FSH injection was given to the women with PCOS twice, i.e. first 15-20 days after a spontaneous period (before progesterone treatment) and second after a 20-days treatment with progesterone per os (after progesterone treatment). The FSH injection was given to the normally cycling women on cycle day 3. After the FSH injection, serum inhibin A and inhibin B levels increased significantly in the women with PCOS but not in the control women. A. a,b,c,e,f p < 0.05, d p < 0.001 B. a,b p < 0.05.

Serum inhibin A levels before the FSH injection were significantly lower in the PCOS women than in the controls (Exp 3) (p < 0.05). Treatment with progesterone suppressed inhibin A levels significantly (Exp 2 as compared to Exp 1) (Figure 2A). After the FSH injection, inhibin A levels increased significantly in the PCOS patients but not in the control women (Exp 3, Figure 2A) and the percentage increase was similar before (353 ± 81%, Exp 1, p < 0.05) and after progesterone treatment (439 ± 124%, Exp 2, p < 0.01). Serum inhibin A levels 24 hours after the FSH injection were significantly lower in the PCOS (Exp 2) than in the controls (Exp 3) (p < 0.05) (Figure 2A).

Serum inhibin B levels were similar in the PCOS and controls before FSH injection (Figure 2B). Progesterone administration did not influence the inhibin B levels. After the FSH injection, inhibin B levels increased significantly in the PCOS women and this was independent of the progesterone treatment (Exp 1 and Exp 2) (p < 0.05 and p < 0.001 respectively) with no significant difference between the two experiments; however, in the controls (Exp 3), inhibin B levels did not increase after the FSH injection (Figure 2B). Serum inhibin B levels after the stimulation with FSH were higher, without reaching significant level, in Exp 1 and Exp 2 than in Exp 3 (p = 0.07) (Figure 2B).

## Discussion

The present results provide new information regarding the control of inhibin secretion in women with PCOS. In accordance with previous studies [3,7,10], serum inhibin A levels were significantly lower in women with PCOS as compared to normally cycling women. Progesterone treatment, apart from normalizing LH levels, further decreased inhibin A levels in the PCOS women. This indicates that LH may contribute to the control of inhibin A secretion in PCOS as it has been suggested for normal women [11]. Nevertheless, based on the specific pattern of low inhibin A and high LH levels in the present study, it is likely that PCOS compared to normal ovaries are less sensitive to endogenous LH stimulus regarding inhibin A secretion.

Our study demonstrates a differential control of inhibin A secretion by FSH in PCOS and in normally cycling women, with the ovaries of PCOS women being more sensitive to FSH stimulus than those of normal women. Although the relatively small number of women may account for the unresponsiveness of inhibin A to FSH in the control group, it is possible that this is firstly related to the methodology used, including the dose and the route of FSH administration, and secondly to the time period of observation. In a previous study with the same number of normal women, in which FSH was not injected sc but i.v., inhibin A levels increased significantly [12] while in another study of a similar sample size, an increase in inhibin A levels was seen in response to purified urinary FSH with the FSH dose being of 200 IU i.m. and only after 36 hours from the injection [24]. However, even a higher number of women would not possibly affect the results, since the small increase in inhibin A levels (≈33% on average) seen in the control group is incomparable to the increase in the PCOS group (≈400%). The increased sensitivity of the PCOS ovaries to FSH found in our study was independent of the preceding effect of progesterone in the context of a simulated luteal phase. This may provide evidence that it is not the lack of regular menstruation that is responsible for the differential response of inhibin A to FSH in the PCOS but this response reflects an intrinsic property of the PCOS ovaries.

The similar circulatory inhibin B concentrations in both PCOS and normal women have been also found in previous studies [3-9]. However, unlike inhibin A, progesterone treatment and concomitant LH levels lowering had no effect on inhibin B levels in the PCOS women suggesting that LH is not probably involved in the control of inhibin B secretion from PCOS ovaries. Relevant data in the literature on the value of LH are scanty and conflicting. Although serum inhibin B levels have been found to be positively correlated to LH levels in vivo [5], hCG administration suppressed inhibin B secretion after 24 hours in PCOS women [8], while it was unable to stimulate inhibin B secretion from polycystic and normal ovaries in vitro [20].

In the present study it was demonstrated that the acute stimulation with FSH increased significantly the serum inhibin B levels in PCOS women something which is in accordance with previous studies [12,21]. Our experiments, in contrast to previous studies, were performed in the proximity of a simulated luteal phase suggesting that the increased sensitivity of the PCOS ovaries to FSH regarding inhibin B secretion is not affected by the preceding action of progesterone. It is therefore likely that, similar to inhibin A, the increased sensitivity of inhibin B to FSH is an intrinsic property of the PCOS ovaries. In contrast to other studies that have shown an increase, the lack of an increase in inhibin B levels in normal women, following FSH administration, is in agreement with a previous study [8]. The difference between them is difficult to explain [9,12,22]. Methodological differences including the route of FSH administration may account for the different results between the present and the previous studies. Whether the relatively higher age of the control as compared to the PCOS women contributed to the ovarian unresponsiveness to FSH in terms of inhibin A and B secretion is not very likely, because both basal FSH and inhibin B levels were comparable in the two groups indicating a similar potential regarding ovarian reserves. Also, despite the slightly higher BMI in the PCOS, similar serum FSH concentrations were achieved in the two groups following the injection of FSH. In addition, if the higher BMI had a negative impact on inhibin B serum levels [23] one would expect lower levels of this protein in PCOS as compared to control women, which was not the case in this study.

In conclusion, our results show for the first time that in PCOS women normalization of elevated LH levels with exogenous progesterone decreased inhibin A levels, while inhibin B levels were unaffected. Besides, following FSH stimulation PCOS ovaries secreted more inhibin A and inhibin B compared to normal ovaries and this was independent of a preceding simulated luteal phase. It is suggested that the PCOS ovaries may be intrinsically less sensitive to endogenous LH regarding inhibin A secretion and more sensitive to exogenous FSH in terms of both inhibin A and inhibin B secretion.

## Competing interests

The authors declare that they have no competing interests.

## Authors' contributions

KD participated in the design and drafted the manuscript, CV performed the experiments, CIM helped to draft the manuscript, SP did the assays, GA helped in the assays, AG helped to draft the manuscript and IEM conceived the study, participated in the design and formulated the final version.

All authors read and approved the final manuscript.
